# One-Step Staining Method for the Identification of Clue Cells and Bacterial Morphotypes Associated with Bacterial Vaginosis

**DOI:** 10.1128/spectrum.01927-21

**Published:** 2022-05-16

**Authors:** Carlos Martínez-Figueroa, Ana Karen Estrada-Moreno, Amalia Vences-Velázquez, Karen Cortés-Sarabia

**Affiliations:** a Laboratorio de Inmunobiología y Diagnóstico Molecular, Facultad de Ciencias Químico Biológicas, Universidad Autónoma de Guerrero, Chilpancingo, Guerrero, Mexico; University of Utah and ARUP Laboratories

**Keywords:** bacterial vaginosis, staining, microscopy, dysbiosis, diagnosis, Amsel, Nugent, clue cells

## LETTER

The vaginal microbiota is a complex ecosystem dominated by *Lactobacillus* spp. (Lactobacillus crispatus, Lactobacillus gasseri, Lactobacillus iners, and Lactobacillus jensenii) and a small number of anaerobic bacteria (1). Bacterial vaginosis (BV) is the main dysbiosis in women of reproductive age (2) and is characterized by diminution in the number of *Lactobacillus* spp. and the overgrowth of anaerobic bacteria such as *Gardnerella*, *Prevotella*, *Atopobium*, *Mobiluncus*, *Bifidobacterium*, *Sneathia*, and *Leptotrichia*, among others (3, 4). Its prevalence rates range from 20 to 60% according to the population studied, with an estimated global incidence of 20 to 30% (5, 6); it is considered one of the main infectious processes at the vaginal level (7). The associated risk factors for the development of BV include multiple sexual partners, the use of a vaginal douche, early onset of sexual activity, alcohol consumption, a smoking habit, age, and race, among others (8, 9). Clinically, it presents in asymptomatic or symptomatic form, and its main signs are the presence of a grayish discharge, a strong “fishy” odor accompanied by a fetid amino odor, and, in rare cases, dysuria and dyspareunia. In the clinical laboratory, the diagnosis of BV can be performed by the use of two different methods, the Amsel criteria and the Nugent score, which allows for the classification of vaginal morphotypes as normal microbiota (scores of 0 to 3), mixed microbiota (scores of 4 to 6), or BV (scores of 7 to 10) (4).

Use of the Amsel criteria represents an easier method for the diagnosis of BV and evaluates clinical characteristics. A positive result is considered when three of the following four established criteria are observed in a vaginal swab sample: the presence of “clue cells,” a grayish vaginal discharge, pH of >4.5, and a positive amine test result (10% KOH) (10). However, despite all of the information regarding the clinical relevance of the early diagnosis of BV, this vaginal condition is usually underdiagnosed or misdiagnosed, due to lack of experience in the correct identification of clue cells (epithelial cells with attached coccobacilli associated with the overgrowth of Gardnerella vaginalis or other anaerobic bacteria, with more than >20% indicating BV) (11) or associated bacteria during the microscopic observation of vaginal swab samples in a physiological saline solution. This problem could be solved by the use of a stain that can be utilized for the adequate identification of cellular structures (clue cells) and bacterial morphotypes; our working group has employed an easy, inexpensive, and fast dye solution that has permitted us to obtain adequate identification of these structures. In addition, this could be useful for personnel with insufficient experience working in a clinical laboratory performing the diagnosis of this condition.

**FIG 1:**
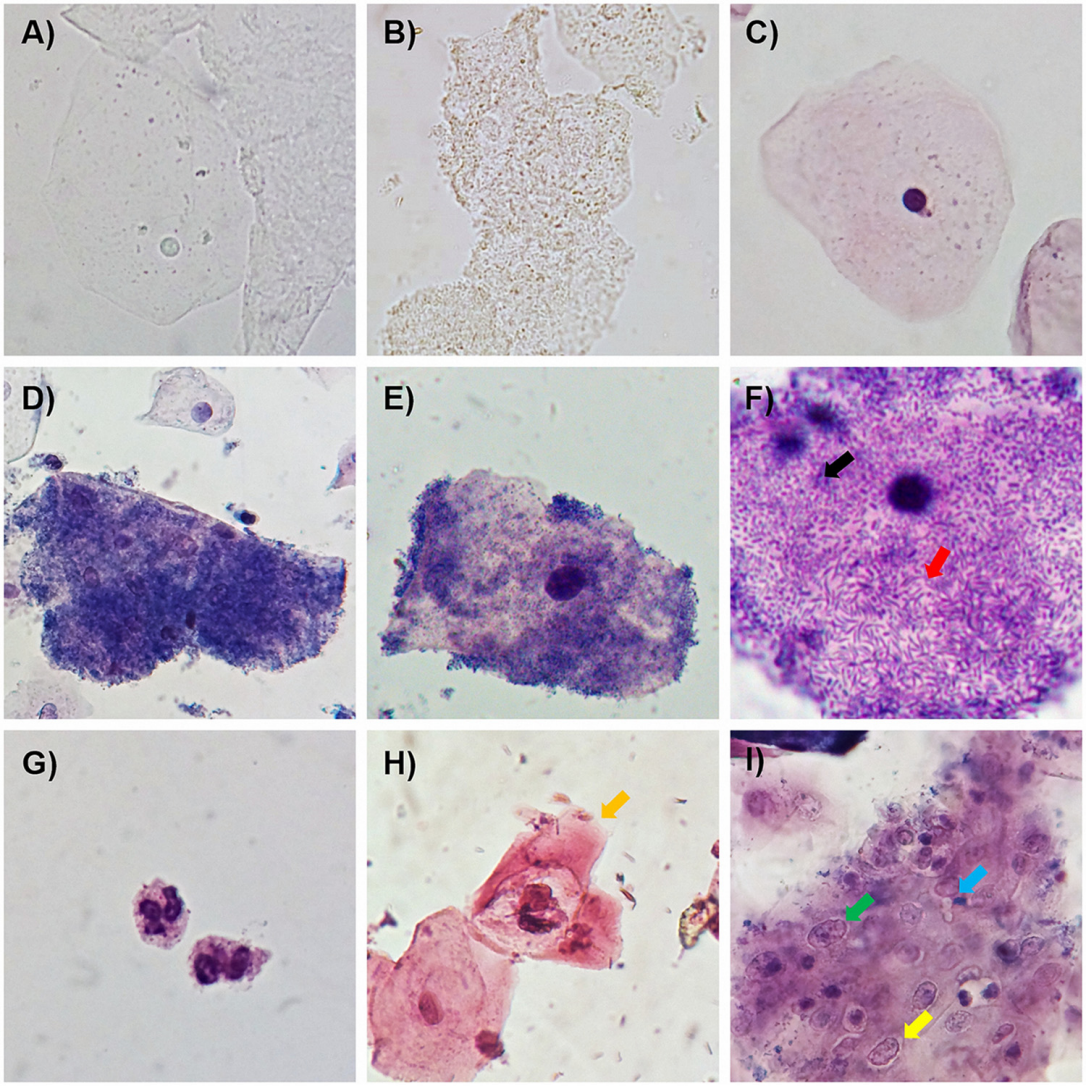
Examples of the usefulness of the A-S dye solution in the diagnosis of BV. (A and B) Fresh observation of a normal squamous epithelial cell (A) and clue cells (B) in a nonstained sample. (C to E) Normal squamous epithelial cells (with normal morphology) and examples of clue cells in samples from patients with BV using the A-S dye solution. (F) Bacterial morphotypes attached to the surface of epithelial cells, including curved bacilli (red arrow) and a coccobacillus (black arrow). (G) Leukocytes (polymorphonuclear). (H) Presumptive koilocyte (associated with HPV infection) with karyomegaly and perinuclear halo (yellow arrow). (I) Group of squamous epithelial cells with inflammation-related changes such as karyomegaly (green arrow) and perinuclear halo (yellow arrow) during *Candida* infection (blue arrow) (1000X amplification).

The dye solution used, called A-S solution, contains Loeffler’s methylene blue and a 0.5% safranin solution, mixed at a ratio of 7:3. Methylene blue-stained bacteria and safranin-stained epithelial cells, in conjunction with the staining of both structures, aid in the adequate identification of clue cells, even at low magnification (magnification of ×100) (Fig. 1A to E). Also, perfect identification and classification of the bacterial morphotypes associated with BV as curved bacilli or coccobacilli (Fig. 1F) attached to squamous epithelial cells and polymorphonuclear leukocytes (Fig. 1G) are possible. In addition, during the time that this dye solution has been employed in our laboratory, we have observed that it can be utilized for the presumptive identification of cellular alterations associated with inflammatory processes and koilocytes related to human papillomavirus (HPV) infection (which needs further analysis) (Fig. 1H and I).

Our pilot study included the evaluation of 1,128 wet preparations of vaginal swab samples to search for clue cells (>20% was considered positive) using the A-S dye solution, and the diagnosis of BV was performed using Amsel criteria and Nugent scores. A total of 93.21% of the patients with BV had >20% clue cells employing the one-step staining method, whereas 98.58% of the healthy patients had <20% clue cells (Table 1). Also, a comparison was performed between the Amsel criteria and Nugent scoring and the observation of clue cells (<20% or >20%). With the Amsel criteria, 97.78% of the subjects with 0, 1, or 2 positive criteria (considered healthy) exhibited <20% clue cells, whereas 95.6% of the patients with 3 or 4 positive criteria (considered to have BV) had >20% clue cells. With the Nugent scoring system, among the patients with <20% clue cells, 76.49% had scores of 0 to 3 (normal microbiota), 21.29% had scores of 4 to 6 (mixed microbiota), and 2.22% had scores of 7 to 10 (BV). In contrast, in the group with >20% clue cells, 95.6% had scores of 7 to 10, which is commonly associated with BV (Table 2).

**TABLE 1:** Relation between clinical diagnosis and the number of clue cells observed using the dye solution

Proportion of clue cells using dye solution	Diagnosis (no. [%])^*a*^	
	Healthy	BV
<20%	836 (98.58)	19 (6.79)
>20%	12 (1.42)	261 (93.21)
Total	848 (100)	280 (100)

a Diagnosis was performed using Nugent scoring and the Amsel criteria.

**TABLE 2:** Comparison of the diagnostic criteria used for BV and the proportion of clue cells observed using the dye solution

Diagnostic finding	No. (%) of samples with proportion of clue cells of^*a*^:		
	<20%	>20%	Total
Amsel criteria			
None	274 (32.05)	0 (0.0)	274 (24.29)
1 or 2	562 (65.73)	12 (4.409	574 (50.89)
3 or 4	19 (2.22)	261 (95.60)	280 (24.82)
Total	855 (100.0)	273 (100.0)	1,128 (100.0)
Nugent score			
0–3	654 (76.49)	1 (0.37)	655 (58.07)
4–6	182 (21.29)	11 (4.03)	193 (16.11)
7–10	19 (2.22)	261 (95.60)	280 (24.82)
Total	855 (100.0)	273 (100.0)	1,128 (100.0)

a The number of clue cells was obtained using the A-S dye solution.

## References

[1] Gupta S, Kakkar V, Bhushan I. 2019. Crosstalk between vaginal microbiome and female health: a review. Microb Pathog 136:103696. doi:10.1016/j.micpath.2019.103696. 31449855

[2] Reiter S, Kellogg Spadt S. 2019. Bacterial vaginosis: a primer for clinicians. Postgrad Med 131:8–18. doi:10.1080/00325481.2019.1546534. 30424704

[3] Chen X, Lu Y, Chen T, Li R. 2021. The female vaginal microbiome in health and bacterial vaginosis. Front Cell Infect Microbiol 11:631972. doi:10.3389/fcimb.2021.631972. 33898328 PMC8058480

[4] Coudray MS, Madhivanan P. 2020. Bacterial vaginosis: a brief synopsis of the literature. Eur J Obstet Gynecol Reprod Biol 245:143–148. doi:10.1016/j.ejogrb.2019.12.035. 31901667 PMC6989391

[5] Bautista CT, Wurapa E, Sateren WB, Morris S, Hollingsworth B, Sánchez JL. 2016. Bacterial vaginosis: a synthesis of the literature on etiology, prevalence, risk factors, and relationship with chlamydia and gonorrhea infections. Mil Med Res 3:4. 26877884 10.1186/s40779-016-0074-5 PMC4752809

[6] Peebles K, Velloza J, Balkus JE, McClelland RS, Barnabas RV. 2019. High global burden and costs of bacterial vaginosis: a systematic review and meta-analysis. Sex Transm Dis 46:304–311. doi:10.1097/OLQ.0000000000000972. 30624309

[7] Onderdonk AB, Delaney ML, Fichorova RN. 2016. The human microbiome during bacterial vaginosis. Clin Microbiol Rev 29:223–238. doi:10.1128/CMR.00075-15. 26864580 PMC4786887

[8] Kamga YM, Ngunde JP, Akoachere J-F. 2019. Prevalence of bacterial vaginosis and associated risk factors in pregnant women receiving antenatal care at the Kumba Health District (KHD), Cameroon. BMC Pregnancy Childbirth 19:166. doi:10.1186/s12884-019-2312-9. 31077161 PMC6511194

[9] Li M, Li L, Wang R, Yan S-M, Ma X-Y, Jiang S, Gao T-Y, Yao Y, Li B. 2019. Prevalence and risk factors for bacterial vaginosis and cervicitis among 511 female workers attending gynecological examination in Changchun, China. Taiwan J Obstet Gynecol 58:385–389. doi:10.1016/j.tjog.2018.11.036. 31122530

[10] Mohammadzadeh F, Dolatian M, Jorjani M, Alavi Majd H. 2014. Diagnostic value of Amsel's clinical criteria for diagnosis of bacterial vaginosis. Glob J Health Sci 7:8–14. doi:10.5539/gjhs.v7n3p8. PMC4802101 25948431

[11] Gardner HL, Dukes CD. 1959. *Hemophilus vaginalis* vaginitis. Ann N Y Acad Sci 83:280–289. doi:10.1111/j.1749-6632.1960.tb40901.x. 13826525

